# Changes to tear cytokines of type 2 diabetic patients with or without retinopathy

**Published:** 2010-12-31

**Authors:** Jingfang Liu, Bingyin Shi, Shuixiang He, Xiaoli Yao, Mark D.P. Willcox, Zhenjun Zhao

**Affiliations:** 1 First Hospital Affiliated to Medical College, Xi’an Jiaotong University, Xi’an, China; 2 Brien Holden Vision Institute, Sydney, Australia; 3 The School of Optometry and Vision Science, University of New South Wales, Sydney, Australia

## Abstract

**Purpose:**

To investigate changes in cytokine levels in tears of type 2 diabetics with or without retinopathy.

**Methods:**

Tears were collected from 15 type 2 diabetics without retinopathy (DNR), 15 patients with retinopathy (DR), and 15 age and gender matched non-diabetic controls. Tear concentrations of 27 cytokines were measured by multiplex bead immunoassay. Cytokine differences between groups, ratios of type-1 T helper (Th1)/type-2 T helper (Th2) cytokines and anti-angiogenic/pro-angiogenic cytokines were analyzed statistically.

**Results:**

The most abundant cytokine detected in tears was interferon-induced protein-10 (IP-10). In comparison with controls, IP-10 and monocyte chemoattracant protein-1 (MCP-1) levels were significantly elevated in DR (p=0.016 and 0.036, respectively) and DNR groups (p=0.021 and 0.026, respectively). Interleukin-1 (IL-1) receptor antagonist (IL-1ra) levels were significantly increased in DNR (p=0.016). Th1/Th2 cytokines interferon-gamma (IFN-γ)/IL-5 and IL-2/IL-5 ratios were significantly increased in DR compared to controls (p=0.037 and 0.031, respectively). Anti-angiogenic/angiogenic cytokines IFN-γ/MCP-1 and IL-4/MCP-1 ratios in DR and DNR were significantly decreased compared to controls (p<0.05). IL-4/IL-8 and IL-12p70/IL-8 ratios were also significantly decreased in DR compared to controls (p=0.02 and 0.045, respectively). No significant correlation was demonstrated between tear cytokine concentrations and glycosylated hemoglobin (HbA1c) or fasting plasma glucose (FPG).

**Conclusions:**

Diabetic tears exhibited elevated levels of IP-10 and MCP-1. The Th1/Th2 cytokine balance may shift to a predominantly Th1 state in DR patients. Pro-angiogenic cytokines are more highly represented than anti-angiogenic cytokines in the tears of diabetic patients.

## Introduction

Diabetes mellitus (DM) can induce various kinds of ocular complications such as diabetic retinopathy (DR), neovascular glaucoma, cataract, refractory deviations, ptosis and palsy of the oculomotorius nerve [1-4]. DR, one of the most common causes of blindness in western populations, is the most frequent diabetic microvascular disease [5]. During the first two decades of disease, nearly all patients with type 1 DM and >60% of patients with type 2 DM develop DR [5].

Although the pathogenesis of DR is yet not completely elucidated, there is increasing evidence that the inflammatory process may play a role. Elevated levels of inflammatory cytokines, chemokines and growth factors can be detected in vitreous fluids and aqueous humor of patients with DR [6-11]. These upregulated inflammatory cytokines and other inflammatory mediators, which may lead to persistent low-grade inflammation and influx of leukocytes, are proposed to contribute to DR-associated damage of the eyes in diabetes patients [12].

The ocular surface is also affected by DM. Reduced corneal sensitivity and altered tear quantity and quality have been reported in diabetic patients [13]. There are significant correlations between the grade of DR and degree of corneal epithelial fragility or corneal sensitivity [14,15]. Dry eye syndrome, an ocular surface disease, is frequently found in diabetic patients. The severity of the dry eye disease has been found to be correlated with the severity of DR [16,17].

Many studies have demonstrated the presence of cytokines in tears. Elevated levels of inflammatory cytokines have been reported in tears from various ocular diseases [18,19]. So far there have been no reports regarding the changes of cytokines in tears of diabetic patients with retinopathy. In the present study we report the results of analysis of 27 cytokines in tears obtained from type 2 diabetic patients with or without retinopathy.

## Methods

### Study subjects

All diabetic patients (hospitalized) and non-diabetic subjects were recruited from the Department of Endocrinology, First Hospital Affiliated, Medical School of Xi’an, Jiaotong University, Xi’an, China. All were ethnic Chinese, residing in the Shaanxi province, China. Before enrolment in the study, all subjects signed an informed consent after explanation of the nature and possible consequences of the study. All experimental protocols were reviewed and approved by the hospital Human Ethics Review Committee and complied with the Declaration of Helsinki for Experimentation on Humans, 1975 and revised in 1983.

DM was diagnosed according to the 1999 World Health Organization (WHO) criteria. DR was diagnosed on the basis of fundus examination and fluorescein angiography. Fifteen type 2 diabetic patients without retinopathy (DNR), 15 patients with retinopathy (DR), and 15 gender- and age-matched non-diabetic controls (with fasting finger blood glucose level below 5.6 mmol/l) were recruited. None of the subjects had clinically detectable ocular infection, age-related macular degeneration, glaucoma or cataract. The duration of diabetes, fasting plasma glucose (FPG) and glycosylated hemoglobin (HbA1c) levels, measured by standard methods used in pathology laboratories, were recorded and no significant differences between DNR and DR groups were found (Table 1).

**Table 1:** Clinical characteristics of diabetic patients and non-diabetic controls.

**Parameters**	**Controls**	**DNR**	**DR**
Sex (M/F)	15 (8/7)	15 (8/7)	15 (7/8)
Age (years)	59.00±2.42	61.27±1.95	61.07±2.16
Diabetic duration (years)	_	8.04±1.74	9.00±1.15
FPG (mmol/l)	_	9.14±0.62	8.24±0.87
HbA1c (%)	_	10.04±0.63	9.89±0.68

In the table, Controls are non-diabetic controls; DR are type 2 diabetic patients with retinopathy; and DNR are type 2 diabetic patients without retinopathy. Data are expressed as mean±SEM.

### Sample collection

Open eye basal tear samples were collected using a blunt glass capillary tube to obtain 5–10 µl tears from the outer canthus of the eyes [20]. Care was taken to minimize stimulation to the eyes to avoid collecting reflex tears. The tear samples were immediately stored at −80 °C until use, except for a period of transportation (20 h) from Xi’an, China to Sydney, Australia. During this time the samples were kept frozen in ice packs.

### Total protein concentrations and immunoglobulin A (IgA) assay in tear samples

Total protein concentrations were assayed in each tear sample using Lavapep protein quantitation kits (Fluorotechnics, Sydney, Australia) and IgA concentration was quantified using an enzyme-linked immunosorbent assay (ELISA; Bethyl Laboratories, Montgomery, AL), each according to manufacturer’s instructions.

### Multiplex analysis of cytokines in tears using the Bio-Plex system

Twenty seven cytokines (interleukin-1β [IL-1β], IL-1 receptor antagonist [IL-1ra], IL-2, −4, −5, −6, −7, −8, −9, −10, −12p70, −13, −15, −17, tumor necrosis factor-alpha [TNF-α], interferon-gamma [IFN-γ], granulocyte-monocyte colony-stimulating factor [GM-CSF], granulocyte colony stimulating factor [G-CSF], platelet-derived growth factor-BB [PDGF-BB], basic fibroblast growth factor [FGF-b], vascular endothelial growth factor [VEGF], MCP-1, macrophage inflammatory protein −1α, −1β [MIP-1α, MIP-1β], EOTAXIN, interferon-induced protein-10 [IP-10], and regulated upon activation, normal T-cell expressed and presumably secreted [RANTES]) in each tear sample were detected using multiplex bead analysis (Bio-Plex Human Cytokine 27-plex panel; Bio-Rad, Hercules, CA). The tear samples were diluted 20-fold using the sample diluent supplied in the kit. Standard curves were generated by using the reference cytokine sample supplied in the kit and were used to calculate the cytokine concentrations in tear samples.

For cytokine recovery rate tests, 133 µl of a 195 fold dilution of the reconstituted cytokine standard stock solution in sample diluent was mixed with 7 µl of a pooled tear sample (20-fold dilution as for other tear samples). The cytokine concentrations in this mixture, in the 195 fold dilution, and in the 20-fold diluted pooled tears in sample diluents were assayed (50 µl/well). The recovery rate for each cytokine was calculated by the following equation:

Recovery rate=A/((B×133+C×7)/140)×100%

In the equation, A is the detected cytokine concentration of the mixture of standard and the tear sample; B is the detected cytokine concentration of the 195 fold standard dilution, C is the detected cytokine concentration of the pooled tear sample.

### Statistical analysis

Data were expressed as mean±standard error (SE) and analyzed using the statistical package for social sciences (SPSS; SPSS Inc., Chicago, IL). The differences between the three groups were assessed by one-way ANOVA with a post-hoc analysis (Bonferroni test). For statistical analysis, variables with skewed distribution (IL-1β, IL-2, IL-4, IL-6, IL-7, IL-9, IL-10, IL-12p70, IL-13, IL-15, EOTAXIN, FGF-b, G-CSF, GM-CSF, IFN-γ, IP-10, MCP-1, MIP-1β, PDGF-BB, and RANTES) were square root transformed; the variables for other cytokines (IL-1ra, IL-5, IL-8, and VEGF) were logarithmically transformed. Differences were considered significant at p<0.05. Correlations between HbA1c or FPG levels in plasma, duration of diabetes and single cytokines or cytokine ratios were calculated by Pearson correlation coefficient. Differences were considered significant at p<0.01.

## Results

### Total protein and IgA levels in tears

In comparison with control subjects (9.4±0.8 mg/ml), tear total protein concentrations in DR (14.8±1.5 mg/ml, p=0.014) and DNR group (13.6±1.3 mg/ml, p=0.044) were significantly elevated. Difference between DR and DNR groups was not significant. No statistical differences were found for tear IgA levels among DR (685.3±56.6 µg/ml), DNR (615.2±54.9 µg/ml), and control groups (531.4±65.0 µg/ml).

### Tear cytokines

To test the effect of the tear matrix on the assay, the recovery rate for each cytokine was measured by adding in the same amount of tear fluid to 195 fold diluted standard solution. The results are listed in Table 2. For 20 of the 27 cytokines the recovery rates were between 80%–120%. The cytokines outside this recovery range were PDGF-BB (66%), IL-12p70 (78%), IL-15 (68%), FGF-b (175%), GM-CSF (53%), MIP-1α (74%), and G-CSF (27%).

**Table 2:** Recovery (%) and positive detection rates (%) for each cytokine in the tear samples and concentrations (pg/ml) of tear cytokines in diabetic patients and non-diabetic controls.

** **	** **	** **	**Concentration (pg/ml)**		
**Cytokines**	**Recovery**	**Positive detection rates**	**Controls**	**DNR**	**DR**
IL-1β	93	100	20.0±2.8	20.9±3.6	16.7±3.2
IL-1ra	113	100	3988.7±685.0	9641.7±1923.9*	7409.8±1506.8
IL-2	91	98	105.1±13.4	108.0±17.1	111.2±14.9
IL-4	95	100	14.6±1.4	15.7±2.2	13.0±1.5
IL-5	100	93	15.0±4.0	10.8±2.6	16.7±8.4
IL-6	90	100	64.7±8.8	67.4±11.3	63.3±12.3
IL-7	84	100	106.9±8.2	117.3±12.9	100.1±8.7
IL-8	93	100	54.3±7.2	64.8±7.6	87.3±26.1
IL-9	103	98	539.4±61.2	658.2±87.4	636.0±107.3
IL-10	97	93	31.6±3.9	35.0±6.1	25.7±5.5
IL-12p70	78	100	142.7±19.9	138.3±27.7	99.2±22.0
IL-13	94	100	40.4±5.3	39.54±7.3	31.0±6.3
IL-15	68	84	25.5±3.2	29.7±5.2	20.1±3.6
IFN-γ	91	100	1463.0±158.8	1612.8±228.2	1957.50±166.1
G-CSF	27	100	324.4±32.2	370.7±49.4	319.4±35.0
GM-CSF	53	80	465.8±42.1	486.9±70.0	455.8±46.1
FGF-b	175	93	355.8±38.0	398.1±53.1	344.6±49.6
PDGF-BB	66	96	532.4±65.7	653.7±96.4	512.2±72.5
VEGF	99	100	221.0±16.7	296.1±22.5	270.7±40.2
EOTAXIN	100	100	178.3±14.1	185.1±20.9	170.1±14.7
IP-10	108	100	10466.7±1178.5	14201.4±835.6*	14410.8±901.3†
MCP-1	90	87	61.0±7.2	91.2±6.6*	92.2±10.4†
MIP-1β	113	96	145.7±15.0	172.8±19.7	205.9±66.0
RANTES	110	100	48.1±5.9	51.0±7.3	35.4±4.6

Data were expressed as mean±SEM. The asterisk indicates a p<0.05 DNR versus Controls and the dagger indicates a p<0.05 DR versus Controls.

When the threshold for cytokine detection was set at mean + (2×SD) of the control (blank) wells, positive detection rates were more than 80% for 24 cytokines (Table 2). The positive detection rates for other 3 cytokines were 20% (IL-17), 27% (TNF-α), and 60% (MIP-1α). Due to the low detection rates/concentrations, these three cytokines were not included in statistical analysis. The most abundant cytokines (mean≥200 pg/ml; Table 2) detected in non-diabetic controls were IP-10 (10,466.7 pg/ml), IL-1ra (3,988.7 pg/ml), IFN-γ (1,463.0 pg/ml), IL-9 (539.4 pg/ml), PDGF-BB (532.4 pg/ml), GM-CSF (465.8 pg/ml), FGF-b (355.8 pg/ml), G-CSF (324.4 pg/ml) and VEGF (221.0 pg/ml). The levels of all other cytokines were below 200 pg/ml (Table 2).

In comparison with non-diabetic controls, IP-10 and MCP-1 levels were significantly elevated in DR (p=0.016 and p=0.036, respectively) and DNR groups (p=0.021 and p=0.026, respectively). IL-1ra levels were significantly elevated in DNR compared to controls (p=0.016). No statistical differences were found between DR and DNR for any cytokines (Table 2).

### Th1/Th2 cytokine ratios in tears

**Figure 1:**
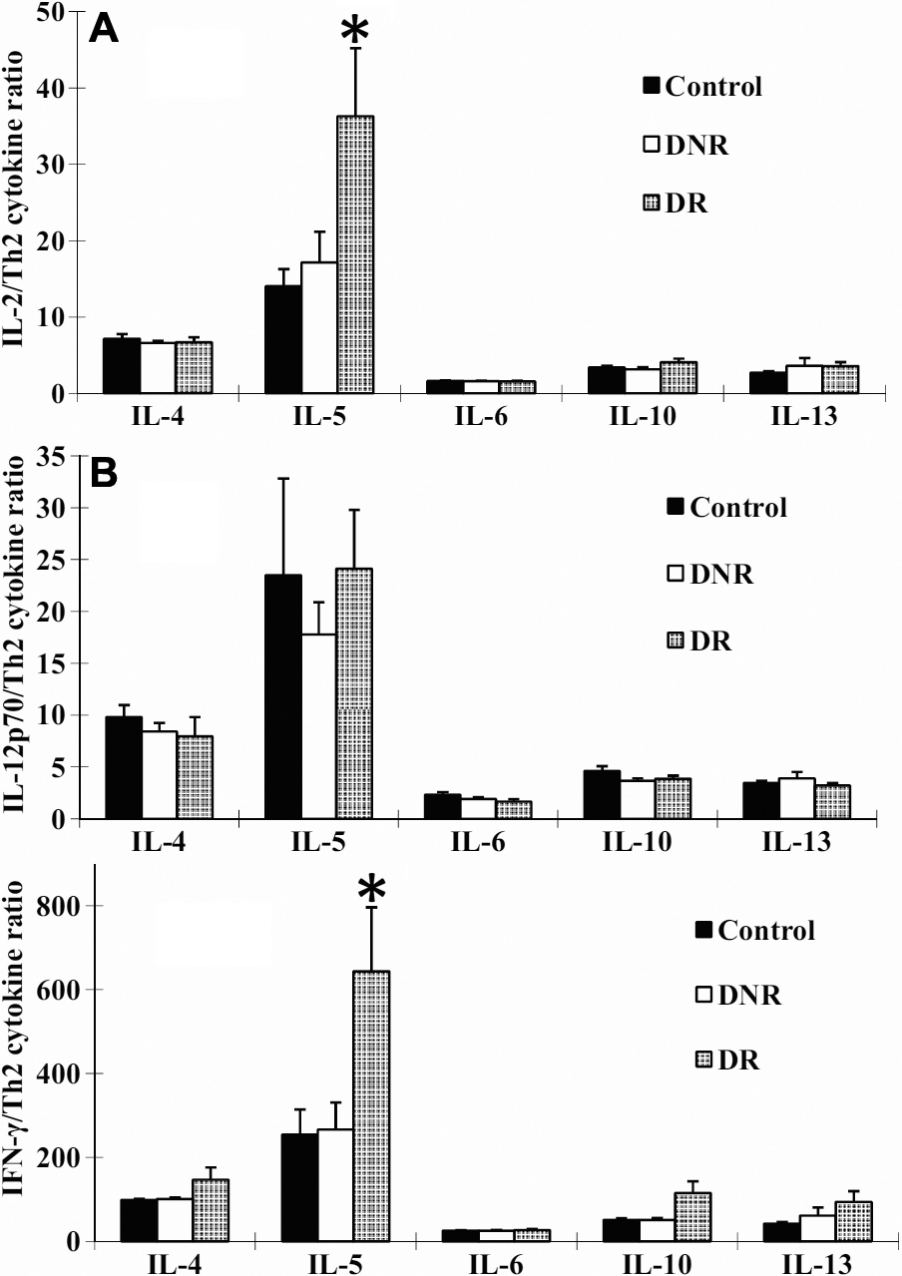
The ratios of Th1/Th2 cytokines in tears of non-diabetic controls, DNR, and DR. Data expressed as mean±SEM **A**: IL-2/ Th2 (IL-4, IL-5, IL-6, IL-10, and IL-13) ratios. **B**: IL-12p70/ Th2 (IL-4, IL-5, IL-6, IL-10, and IL-13) ratios. **C**: IFN-γ/ Th2 (IL-4, IL-5, IL-6, IL-10, and IL-13) ratios. The asterisk indicates a p<0.05 versus non-diabetic controls.

Ratios between Th1 cytokines (IL-2, IL-12p70, and IFN-γ) and Th2 cytokines (IL-4, IL-5, IL-6, IL-10, and IL-13) in tears were calculated (Figure 1). IL-2/IL-5 and IFN-γ /IL-5 ratios were found to be significantly higher in DR (36.3±8.9 and 643.8±152.5, respectively; Figure 1) compared to those in non-diabetic controls (14.1±2.2, p=0.031 and 254.45±60.1, p=0.037, respectively). These ratios in DNR were not significantly different to either non-diabetic controls or DR subjects. No significant differences were observed in the ratios of IL-2/IL-4, IL-2/IL-6, IL-2/IL-10, IL-2/IL-13, IL-12p70/IL-4, IL-12p70/IL-5, IL-12p70/IL-6, IL-12p70/IL-10, IL-12p70/IL-13, IFN-γ/IL-4, IFN-γ/IL-6, IFN-γ/IL-10, or IFN-γ/IL-13 among the three groups.

### Ratios of anti-angiogenic and pro-angiogenic cytokines in tears

**Figure 2:**
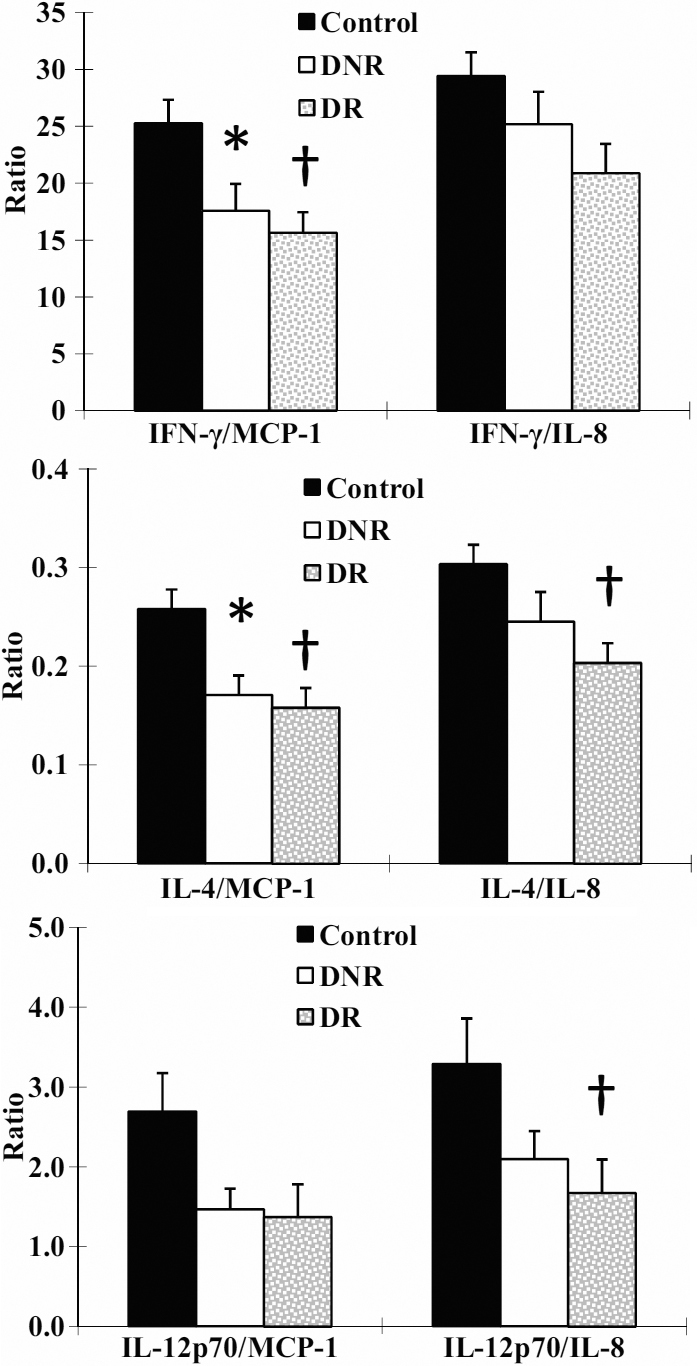
The ratios of anti-angiogenic cytokines (IFN-γ, IL-4, or IL-12p70) and angiogenic cytokines (MCP-1, IL-8) in tears of non-diabetic controls, DNR, and DR. Data was expressed as mean±SEM. The asterisk indicatesd a p<0.05 DNR versus non-diabetic controls and the dagger indicates a p<0.05 DR versus non-diabetic controls.

To explore the anti-angiogenic/pro-angiogenic cytokine profiles, the ratios of each potentially anti-angiogenic cytokine (IL-1ra, IP-10, IFN-γ, IL-4, IL-10, IL-12p70, and IL-13) to each potentially pro-angiogenic cytokine (IL-1β, MCP-1, EOTAXIN, RANTES, IL-8, VEGF, FGF-b, G-CSF, GM-CSF, PDGF-BB, IL-2, IL-6, and IL-15) in tears were calculated. The ratios of IFN-γ/MCP-1, and IL-4/MCP-1 in DR (15.64±1.81 and 0.16±0.02) and DNR (17.58±2.38 and 0.17±0.02) were significantly decreased compared with those in non-diabetic controls (25.26±2.14 and 0.26±0.02, both p<0.05), indicating a potentially pro-angiogenic environment in the ocular surface of the diabetic patients. The ratios of IFN-γ/IL-8, IL-4/IL-8, and IL-12p70/IL-8 were decreased in DR compared with those in non-diabetic controls (20.89±2.59 versus 29.44±2.08, p=0.06; 0.20±0.02 versus 0.30±0.02, p=0.02 and 1.67±0.42 versus 3.29±0.57, p=0.045, respectively), again indicating a potentially pro-angiogenic environment. These results are summarized in Figure 2. No differences were found for ratios of the other anti-angiogenic and angiogenic cytokines among the three groups.

### Correlation between cytokine profiles and disease phenotype

Correlation analysis showed that IL-2, IL-4, IL-7, EOTAXIN, and IFN-γ concentrations in tears were negatively correlated with duration of diabetes, i.e., the shorter the duration of diabetes the higher the cytokine concentration. The correlation coefficients and p values are summarized in Table 3. There were no other significant correlations for the analysis using single cytokine levels or the ratios. No correlation was demonstrated between tear cytokine concentrations and serum HbA1c or FPG levels.

**Table 3:** Correlations between concentration of tear cytokines and duration of diabetes.

**Cytokines**	**Correlation coefficients**	**p values**
IL-2	−0.518	0.003
IL-4	−0.474	0.008
IL-7	−0.478	0.008
EOTAXIN	−0.477	0.008
IFN-γ	−0.474	0.008

## Discussion

There is much debate from various studies regarding cytokine levels in human tears [21-24]. Whether these inconsistencies result from the use of subjects of different ethnicities (and perhaps diet), age, and/or different detection methods remain to be investigated. In this study, multiplex bead immunoassay was used because of its capacity to quantify multiple cytokines simultaneously in samples of very small volumes, such as tears. No other techniques can measure so many cytokines in individual basal human tear samples. Some of the detected cytokine concentrations in our non-diabetic controls are in agreement with the levels observed by other researchers using similar multiplex bead analysis technique [22,25-27]. As in other studies, some inconsistencies were also found compared with these studies, i.e., we observed a higher level of PDGF-BB, IFN-γ, FGF-b, and lower VEGF levels compared to theirs. From our experience, the absolute concentration of a given cytokine may change between different assay techniques, but the trend of change in expression of each cytokine under different pathological conditions remains the same. Inconsistencies in absolute values should not compromise the relative comparisons of individual cytokines and their ratios among the different groups.

The cytokine recovery test of this assay showed that the tear matrix did not interfere with the detection of most the cytokines. Twenty out of the 27 cytokines could be reliably detected in tears using this technique (recovery rate between 80%–120%). The recovery rates for all other cytokines, except for G-CSF, were between 53%–175%, which is also acceptable. The only cytokine that could not be reliably detected was G-CSF, with a recovery rate of 27%. Since this cytokine did not show significant changes between the groups, this assay defect should not compromise the significance of this work.

It is a well known fact that collection technique can influence the protein profile of a tear sample. Secretory IgA is a constitutive tear protein and its level in non-stimulated (open-eye or basal) tears is significantly higher than that in stimulated (or reflex) tears, possibly due to the dilution effect as a result of increased tear flow rate [28]. In the present study significant differences were found for tear protein concentrations between diabetic patients (with or without retinopathy) and non-diabetic controls, but no difference for IgA levels among the three groups was detected. These results may indicate that our tear collection technique does not cause the production of reflex tears, or the level of reflex tear production caused by the capillary tubes was limited and comparable among the subjects/groups. Thus the increased levels of tear proteins in diabetic patients are not due to the tear collection technique and may result from some pathological condition, such as leakage of blood components caused by a sub-acute inflammatory reaction.

To the best of our knowledge, this is the first study to measure 27 cytokines simultaneously in the tears of diabetic patients. Significantly elevated tear MCP-1 and IP-10 levels in DR and DNR, and IL-1ra levels in DNR were found compared to those in non-diabetic controls. Pro-inflammatory cytokines IP-10 and MCP-1 were also reported to be elevated in the vitreous of patients with DR [10,11,29]. IL-1ra is an anti-inflammatory factor that inhibits the pro-inflammatory activity of IL-1β by competitively binding to the type 1 IL-1 receptor without triggering signal transduction [30]. Several studies have suggested that an increase in IL-1ra production could be a compensatory response to immuno-inflammatory activation present at diabetic patients [31,32]. Diabetic patients experience damaged corneal epithelium and basement membranes due to abnormal glucose metabolism, chronic hypoxia, and altered immune status at the ocular surface [33,34].

Th1 cytokines stimulate cell-mediated immunity, and promote inflammation and cytotoxicity. While Th2 cytokines assure regulatory function, thus mediate protective function during diabetes. In the current study, individual Th1 or Th2 cytokine levels were not significantly different among the three groups. However, Th1/Th2 cytokine ratios of IFN-γ/IL-5 and IL-2/IL-5 were significantly higher in DR than in non-diabetic controls or DNR, suggesting that secretions of Th1 and Th2 cytokines in tears of DR patients were shifted to a Th1-dominant state. These data support the hypothesis that Th1 cytokines predominate and create an inflammatory response in the pathogenesis of DR.

The two chemokines elevated in the tears of diabetics, IP-10 and MCP-1, are potentially anti-angiogenic and pro-angiogenic factors, respectively [35,36]. In addition, IFN-γ, IL-4, and IL-12p70 have been reported to be anti-angiogenic, while IL-8 is an angiogenic factor [37,38]. In the present study, the ratios of IFN-γ/MCP-1 and IL-4/MCP-1 were decreased in patients with or without DR, and IFN-γ/IL-8, IL-4/IL-8, and IL-12p70/IL-8 ratios were also decreased in DR. These results suggest a pro-angiogenic environment in the tears of diabetics, especially those with retinopathy. Aberrant angiogensis can lead to formation of tube-like structures and vascular hyperpermeability, resulting in leakage of plasma proteins. These proteins participate in extracellular matrix remodelling and tissue destruction, and play a key role in inflammatory process at ocular surface of diabetic patients [39].

IL-12 possesses anti-angiogenic activity, mediated by stimulation of T-helper lymphocytes and induction of IP-10 expression [38]. Conversely, TNF-α is a pro-angiogenic mediator [40]. Disequilibrium between these pro- and anti-angiogenic cytokines can promote late diabetic complications [41]. Previous studies have shown that aging is associated with increased levels of circulating TNF-α and reduced production of IL-12 [42-45]. In this study, the age range of the patients was too narrow to analyze for correlations. No correlations were observed between the concentrations of tear cytokines and HbA1c or FPG in diabetic groups. This suggests that inflammation at the ocular surface may not be directly correlated to changes in metabolism commonly associated with diabetes.

An interesting finding in this study is that certain tear cytokines exhibited a negative correlation with duration of diabetes. These results may indicate that in the initial stages of diabetes there are major changes in cytokine concentrations. However, these changes seem to even out over time. Netea et al. [45] reported an increase in plasma IL-1 and a decrease in IL-1ra/IL-1 ratio in newly diagnosed type 1 diabetic patients, whereas IL-1 production in long-standing type 1 diabetic patients did not differ from the control group. However, it should be borne in mind that the duration of diabetes may not accurately reflect their true history of diabetes. Diabetes can often go under detected for several years [46].

The diabetic condition affects the eye in many different ways due to peripheral neuropathy and poor metabolic control, which can result in dry eye, meibomian gland dysfunction, reduction of corneal sensibility and other deleterious characteristics [37,47,48]. Any of these complications can change cytokines profile in tears. Further studies using more defined patient groups are needed to clarify the contribution of each of these ocular complications to the changes in cytokine expression observed. While some differences in three cytokine levels were found between the groups, these changes were less than twofold, lower than the changes reported in other body fluid [49]. The changes in Th1/Th2 cytokine ratios or anti-angiogenic/pro-angiogenic cytokines ratios were also relatively low. These results may indicate that the ocular surface is not very vulnerable to this destructive disease.

One limitation of our study is that the majority of patients in DR group had non-proliferative DR (NPDR; 11 out of 15 patients). This may be the reason that no differences were found between DNR and DR for any of cytokine concentrations. A previous study demonstrated that, in comparison with DNR, there were no significant changes in tear film and ocular surface in patients with NPDR, but significant changes were found in patients with proliferative DR (PDR) [47].

In summary, we detected elevated pro-inflammatory cytokines MCP-1 and IP-10, and a compensatory increase in anti-inflammatory cytokine IL-1ra in tears of diabetic patients with and without retinopathy. IFN-γ/IL-5 and IL-2/IL-5 ratios were increased in tears of DR patients. Decreased ratios of anti-angiogenic and angiogenic cytokines IFN-γ/MCP and IL-4/MCP-1 in patients with and without DR, and IFN-γ/IL-8, IL-4/IL-8, and IL-12p70/IL-8 in DR patients were also observed. These results demonstrate that chronic inflammatory reaction and angiogenesis may occur at the ocular surface of diabetic patients. Further studies are required to define the therapeutic potential of cytokine-based interventions in preventing DR or other diabetic ocular complications.
